# Economic burden of diabetic foot ulcer: a case of Iran

**DOI:** 10.1186/s12913-024-10873-9

**Published:** 2024-03-21

**Authors:** Reza Hashempour, SeyedHadi MirHashemi, Fariba Mollajafari, Soheila Damiri, Ali ArabAhmadi, Behzad Raei

**Affiliations:** 1 Department of Health Economics and Statistics, Vice-Chancellor’s Office in Treatment Affairs, Shahid Beheshti University of Medical Sciences, Tehran, Iran; 2 Department of General Surgery, Loghman Hakim Hospital, Shahid Beheshti University of Medical Sciences, Tehran, Iran; 3 Health Economics, Management, and Policy Department, Virtual School of Medical Education & Management, Shahid Beheshti University of Medical Sciences, Tehran, Iran; 4 Department of Public Health, Qaen School of Nursing and Midwifery, Birjand University of Medical Sciences, Birjand, Iran; 5 Razi Educational and Therapeutic Center, Tabriz University of Medical Science, Tabriz, Iran

**Keywords:** Diabetic foot, Cost, Economic burden, Iran

## Abstract

**Background:**

Diabetic foot ulcer (DFU) is known as a serious complication of diabetes mellitus in patients with diabetes, imposing heavy medical costs on healthcare systems due to its chronic nature. patients with severe diabetic foot ulcer are often disabled to work, and some of them may even die, leading to associated productivity losses. Since no previous study has investigated the economic burden of DFU in Iran, this study is to estimate the economic burden of diabetic foot disease in Iran.

**Methods:**

In this descriptive cross-sectional study, randomly selected samples consisted of 542 patients with DFU, hospitalized in the hospitals of Shahid Beheshti University of Medical Sciences. The demographic profile and cost data used in this analysis were derived from a researcher-designed checklist. Lost productivity was calculated based on Human Capital Approach, and the total economic cost of DFU was determined using patient-level data on costs and prevalence data from the global burden of diseases reports. All analyses were performed using SPSS software (Version 23), and Microsoft Excel (Version 19).

**Results:**

The economic burden of DFU in Iran in two scenarios of discounting future costs and not discounting them was about $8.7 billion and $35 billion, respectively (about 0.59 and 2.41% of GDP). 79.25% of the estimated costs in this study were indirect costs and productivity losses, of which 99.34% (7,918.4 million Dollars) were productivity losses due to premature death. 20.75% (2,064.4 million dollars) of the estimated costs in this study were direct costs. The average length of stay (LOS) was 8.10 days (SD = 9.32), and 73.3% of patients recovered and were discharged after hospitalization and 7.6% died. The majority of the costs are imposed on the age group of 60–69 year (53.42% of the productivity lost due to hospital length of stay, 58.91% of the productivity lost due to premature death & 40.41% of direct costs).

**Conclusions:**

DFU represents a heavy burden to patients, Iran’s health system, and the economy. Early prevention strategies need to be prioritized in making public health policies. These policies and decisions can be in the area of changing lifestyle, health education, changing people's behavior, and encouraging physical activity that targeted high-risk populations in order to reduce the prevalence of diabetic foot and resulting substantial economic burden.

## Background

Diabetes mellitus is a conundrum concerned with public health, which is caused by defects in carbohydrate, protein, and lipid metabolism resulting from abnormalities of insulin secretion, insulin resistance secretion, or both [1]. The global prevalence of this disease, as one of the commonest types of chronic metabolic disorders [2], is between 3 and 13% [3]. In Iran, the prevalence of diabetes in the age group 25–70 years has been reported to be 11.9%, while most people are not aware of their disease [1]. In Iran, over 8.6% of the total health expenditures are spent on diabetes, compared to 11.6% of health spending on it worldwide. Diabetic patients have 4–5 times higher hospitalization rates, 2.6 times more annual visits, and 2.5 times more prescription volume than non-diabetics [4]. The incidence of this disease continues to increase annually [2, 4] and the number of diabetics around the world is predicted to reach 366 million by 2030.

Nephropathy and foot ulceration are among the most severe and chronic complications of diabetes mellitus [2], increasing the risk of mortality and morbidity and economic burdens as well [5]. DFU is a serious and frequent complication of diabetes mellitus that manifests itself with deep wounds. The contributing factors of DFU include peripheral sensory neuropathy, foot deformity, minor foot trauma, and peripheral arterial disease [6, 7]. Diabetic ulcers occur in patients as a consequence of poor blood supply after developing diabetes [8]. Treatment regimens include nutritional management, physical activity, hypoglycemic medication, and insulin therapy [9]. Many wounds remain unhealed for months, some never heal, and some wounds are amputated [10]. These nonhealing wounds may result in damage to body organs or even deadly and severe infections for the patient. Therefore, antibiotic therapy plays a leading role in reducing these complications [8]. Half of the patients with diabetic foot ulcers suffer from a wound so 17% of them need to undergo foot amputation [11]. It is estimated that the 5-year mortality in amputees after the first amputation ranges from 68 to 79%, and the 5-year mortality after DFU is 40% [12]. Among diabetics, 2% to 5% will develop DFU annually, and the risk of foot ulcers during their lifetime varies from 15 to 25% [3]. The prevalence of DFU in Iran was estimated to be 6.4% [13]. This disease poses a heavy financial and psychological burden on patients and their families as well as the health system [8]. Healthcare costs for diabetic patients with DFU are 5 times higher than those for non-DFU diabetics [5]. The per capita cost of treating a diabetic foot ulcer in America is $17,500, and the total costs of managing a diabetic foot ulcer in England amount to $1.32 billion [14]. Diabetic foot accounts for 10.7% of the total cost of diabetes complications [15], and the results of international studies have shown that the annual mean direct cost of treatment was $1399 per patient [10] and the median direct cost was $1023 [10]. The burden of diabetic ulcers in Iran was estimated to be 5848 years [16]. Some studies found that indirect costs comprised a significant proportion of the economic burden. The cost of medications allocates a larger proportion of the direct costs, whereas wages lost from work while seeking treatment and being treated constitute the main portion of indirect costs. The high prevalence of the disease in developing countries, including Iran, has posed a challenge to disease prevention, early or timely diagnosis, and appropriate treatment. Reliable evidence on the economic burden of disease is required to assist local decision-makers and health policymakers to make informed choices in optimal allocation and efficient use of resources. In Sweden, for instance, a particular health care program at the population level for 150,000 people using a wound healing center, multidisciplinary care, and continuous education, has reduced the annual costs of wound care up to SEK 6.96 million over 10 years [17, 18]. In Iran, no study has yet estimated the economic burden of DFU, hence this study was conducted to estimate the economic burden of diabetic foot ulcers in Iran.

## Methods

### Data and study population

Cost of Illness (COI) studies aim to identify and measure all costs of disease. They describe and estimate the economic burden of a particular disease for society and therefore reflect the savings that could be made if the disease would be eradicated [19]. COI studies are categorized as descriptive studies and include the identification and valuation of cost items to determine the total costs of a specific condition/disease and its economic burden. This cross-sectional descriptive study was conducted with the aim of estimating the cost of diabetic foot ulcers in Iran from the perspective of society [20]. The data required for the estimates in this study were collected from the population of patients referred to hospitals affiliated to Shahid Beheshti University of Medical Sciences in Tehran in 2021. The data of 541 patients with DFU who were selected using random sampling method were considered. Patients were selected if they had traveled to the hospital and had been diagnosed with DFU documented in their medical records. Traditionally, in COI studies, costs are stratified into direct medical, direct non-medical and indirect costs. The method of estimating these costs is explained in following sub-headings.

### Direct costs

Direct costs are patient care costs and include direct medical costs and direct non-medical costs. In various studies, several items have been listed or calculated in the subcategory of direct costs. For example, Yousefi et al. [21] have identified 39 items for direct costs. In this study, the data of the selected sample was collected using an electronic form that had sections to record demographic information and hospital bill details. In this study, the data of the selected sample was collected using an electronic form that had sections to record demographic information and hospital bill details. Direct medical costs were calculated based on these data. In order to estimate the direct non-medical costs, the contact information of the patients recorded in their medical records was extracted, and the data related to the direct non-medical costs, including transportation costs, etc., were asked from them. From the sum of direct medical and non-medical costs, the total direct costs of each patient were calculated. In order to investigate the effect of different factors on the difference in average costs, Mann–Whitney and Kruskal–Wallis statistical tests were used in SPSS version 21. The direct costs imposed on the entire population were calculated as follows.

$$\mathrm{DC}=\sum{ADC}_{a_i}\times{Prevalence}_{{DMT2}_{a_i}}\times DFU\;rate$$

DC: Direct Cost

${ADC}_{{a}_{i}}$: Average Direct Cost in age group i

${Prevalence}_{{DMT2}_{{a}_{i}}}$: Prevalence of type 2 diabetes in age group I, The prevalence of diabetes by age group in Iran was extracted from the global burden of diseases reports in 2019 [22].

${DFUrate}_{{a}_{i}}:$ Prevalence of DFU in diabetic patients in the age group i. According to the results of a systematic review by Zhang et al., the prevalence of diabetic foot ulcers in type 2 diabetes was about 6.4% (95%CI: 4.6–8.1%) [23]. Other studies have reported different prevalence rates [13, 24]. By combining the prevalence rates of diabetes in the country and the multiple prevalence rates of DFU in diabetic patients that were extracted from studies, several scenarios were developed and finally the average and upper and lower limits for the direct costs imposed on Iran due to DFU was calculated.

### Indirect costs

Indirect costs in COI studies refer to the loss of productivity due to mortality and morbidity that is imposed on the individual, family, society and employer and includes lost productivity as a result of premature death, morbidity and impairment, absenteeism, foregone leisure times and the time spent by Siren's family to meet [25]. Yousefi and colleagues have identified 10 items for this cost group [21]. In this study, to estimate the loss of productivity due to premature death, the age distribution data of sample patients obtained from Shahid Beheshti University of Medical Sciences who died were separated from the total data. Productivity loss due to death for each deceased patient was calculated as follows.

$${PLpd}_i={{DR}_I\times\mathrm{Expectation}\;\mathrm{of}\;\mathrm{life}}_i\times{LFP}_i\times annual\;wage\times{Prevalence}_{{DMT2}_{a_i}}\times{DFUrate}_{a_i}$$

PLpd: Loss of productivity due to premature death

${DR}_{I}$: Death rate: Death rate in age group r based on sample data

$\mathrm{Expectation}\;\mathrm{of}\;{\mathrm{life}}_i$: Expectation of life at age i, which is extracted from the life tables provided by the World Health Organization [26].

${LFPR}_{i}$: The data of the labor force participation rate by age groups was extracted from the reports of Iran Statistics Center [27].

$wage$: The average annual salary and benefits of a worker in 2021 in Iran [28].

Considering that the loss of productivity caused by death is distributed in different future years, these costs are calculated and presented with two approaches, undiscounted and discounted at a rate of 5%.

The loss of productivity due to staying in the hospital was also calculated as follows:

$${PLh}_i={\sum({ALOS}_I}\times{LFP}_i\times daily\;wage\times{Prevalence}_{{DMT2}_{a_i}}\times{DFUrate}_{a_i})$$

${ALOS}_{I}$: The average length of stay in the hospital for each age group, which was extracted from the sample data obtained in the hospitals of Shahid Beheshti University of Medical Sciences.

$${INDC}_{i}={PLpd}_{i}+{PLh}_{i}$$

### Intangible cost

Intangible costs represent the pain and suffering of a patient due to the disease, which is mainly evaluated by the reduction in the quality of life [29]. Intangible costs have different dimensions, so its measurement is complicated. For example, Yousefi et al. [21] identified 44 items in the group of intangible costs, and considering the various considerations that can be made in calculating intangible costs, in this study, the focus was only on direct and indirect costs.

Therefore, the total economic burden of diabetic foot ulcers was estimated as follows:

$$Total\;CoI=\sum({DC}_i+{INDC}_i)$$

#### Unit cost

Since all the estimated costs were based on Iranian Rials, in order to make it possible to compare the costs with other studies, they were converted based on conversion rate of Iranian Rial to US$ in 2021 (42000 IR = 1 US$). The economic burden of DFU is expressed through two scenarios of discounting future costs and not discounting them.

#### Data analysis

Data were analyzed using Excel software version 2021 and Stata software version 17. Both descriptive and analytical statistics were utilized. Furthermore, one way sensitivity analysis was conducted to assess the effects of key variables on total cost.

## Results

In total, the electronic records of 541 patients with diabetic foot admissions to the hospitals affiliated with Shahid Beheshti University of Medical Sciences were included for analysis. Of the cohort of patients, 68.2% were males aged between 60 and 69. Most of the patients (54.3%) were in employment and Iranian (97.2%). The average length of stay (LOS) was 8.10 days (SD = 9.32), and 73.3% of patients recovered and were discharged after hospitalization (Table 1).

**Table 1:** Frequency and cost of variables (US dollars)

Variable	Subgroups	Frequency		Mean	SD	Median	Interquartile	*P*-value
		**N**	**%**					
**Gender**	Male	369	68.2	2529.50	3738.12	1570.46	2316.87	0.62
	Female	172	31.8	2694.76	3405.74	1596.02	2996.51	
**Age**	< 40	29	5.4	3326.88	5333.95	1311.34	3346.56	0.68
	40–49	94	17.4	2389.57	3208.20	1546.85	1689.63	
	50–59	151	27.9	2371.05	2778.08	1482.43	2344.04	
	60–69	161	29.8	2726.33	4593.60	1580.68	2497.82	
	$\ge$ 70	106	19.6	2630.35	2779.72	1771.72	3289.59	
**Job-status**	Active involvement	294	54.3	2258.64	2712.47	1386.70	2035.70	0.03
	Passive involvement	247	45.7	2966.98	4465.68	1798.72	1798.72	
**Nationality**	Iran	526	97.2	2601.84	3669.35	1579.87	2410.87	0.45
	Afghan	15	2.8	1887.87	1941.55	970.92	2310.94	
**Discharge state**	Recovered	396	73.3	2735.68	3757.28	1759.04	2350.47	*P* < 0.001
	DAMA	87	16.1	930.90	1371.51	307.46	1226.88	
	Death	41	7.6	4318.64	6434.57	2752.39	6004.90	
	Other	16	3	3295.63	3197.01	1861.1	4809.07	

*DAMA* Discharge against medical advice

Table 2 provides a summary of estimated direct medical costs associated with DFU.

**Table 2:** Categorized direct costs of DFU

Cost sub-category	percent	Mean (SD)	Confidence interval		Median (IQR)
			Lower limit	Upper limit	
Visit and consultation	8.60	221.81(320.91)	194.71	248.92	135.41(225.34)
Bed	29.54	762.70(1056.58)	673.46	851.93	453.62(678.02)
Diagnosis	16.95	437.72(646.44)	383.12	492.31	264.81(425.26)
Consumed drug and tool	30.41	785.22(1141.75)	688.80	881.65	455.01(715.76)
Hoteling	1.66	42.79(60.73)	37.66	47.91	25.11(38.97)
Operation room and surgery	9.02	233.11(372.42)	201.65	264.56	146.68(217.88)
Non-medical direct costs	3.82	98.67(159.18)	85.22	112.11	56.19(98.42)
Total direct cost	100	2582.04(3633.41)	2275.18	2888.90	1575.04(2415.01)

The total cost recorded in the medical files for DFU patients aggregated $1,396,886, the minimum cost belonged to nursing services with an average of $79 (1.66%), and the highest one so to drug and consumable services averaging $424,808 (30.41%). The analysis showed a significant association of direct medical cost with job status (*P* = 0.03) and discharge states (*P* < 0.001). No significant relationship was detected for other variables. Our results indicate that direct medical costs were significantly lower for employed patients compared with unemployed patients. Regarding discharge states, post hoc Scheffe tests revealed that the patients discharged against medical advice had a lower direct cost than the patients who died or recovered (*P* < 0.05). We also found a positive correlation between the length of stay and the total cost of hospitalization (*r* = 0.58, *P* < 0.001). However, no relationship exists between the length of stay and age (*r* = 0.07, *P* = 0.07). The findings suggest a weak link may exist between job status and discharge state, so 75.3% of employed patients were discharged after recovery and 13.4% of them had DAMA, while, this figure in unemployed patients was 71.7% and 18.4%, respectively.

DFU with a total cost between $30 billion and $39 billion – a mean of $35 billion – imposes a heavy burden on the Iranian population. Without discounting, the mean direct cost and mean costs resulting from the lost productivity of DFU were estimated at $1.8 billion, and $34 billion, respectively, representing 5% and 95% of total costs attributable to DFU (Table 3).

**Table 3:** Economic burden of DFU (Million $ US)

Cost category			Value	Age groups					
				< 40	40–49	50–59	60–69	≥ 70	Total
Direct costs			Lower	105.1	132.0	279.1	603.4	431.8	1,551.4
			Mean	114.9	140.0	303.0	730.6	519.4	1,807.9
			Upper	124.8	148.0	326.8	857.8	607.1	2,064.4
Indirect costs	Hospital stay productivity lost		Lower	2.6	4.7	8.5	20.2	3.5	39.6
			Mean	2.9	5.0	9.2	24.5	4.2	45.8
			Upper	3.1	5.3	10.0	28.7	4.9	52.0
	Productivity lost due to premature death	Not discounted	Lower	1,535.5	4,866.0	9,191.9	10,668.1	3,048.4	29,309.9
			Mean	1,679.5	5,160.6	9,978.7	12,919.2	3,667.1	33,405.1
			Upper	1,823.5	5,455.2	10,765.6	15,170.4	4,285.7	37,500.2
		Discounted (5%)	Lower	8.0	297.4	842.6	3,340.2	1,313.4	5,801.6
			Mean	8.7	315.2	914.8	4,041.4	1,579.9	6,860.0
			Upper	9.5	333.1	986.9	4,742.6	1,846.4	7,918.4
Total cost of illness	Not discounted		Lower	1,643.2	5,002.7	9,479.5	11,291.6	3,483.7	30,900.9
			Mean	1,797.3	5,305.6	10,290.9	13,674.2	4,190.7	35,258.7
			Upper	1,951.4	5,608.5	11,102.3	16,056.8	4,897.6	39,616.6
	Discounted (5%)		Lower	115.7	434.1	1,130.3	3,963.7	1,748.7	7,392.5
			Mean	126.6	460.3	1,226.9	4,796.4	2,103.5	8,713.7
			Upper	137.4	486.4	1,323.6	5,629.0	2,458.4	10,034.8

**Fig. 1:**
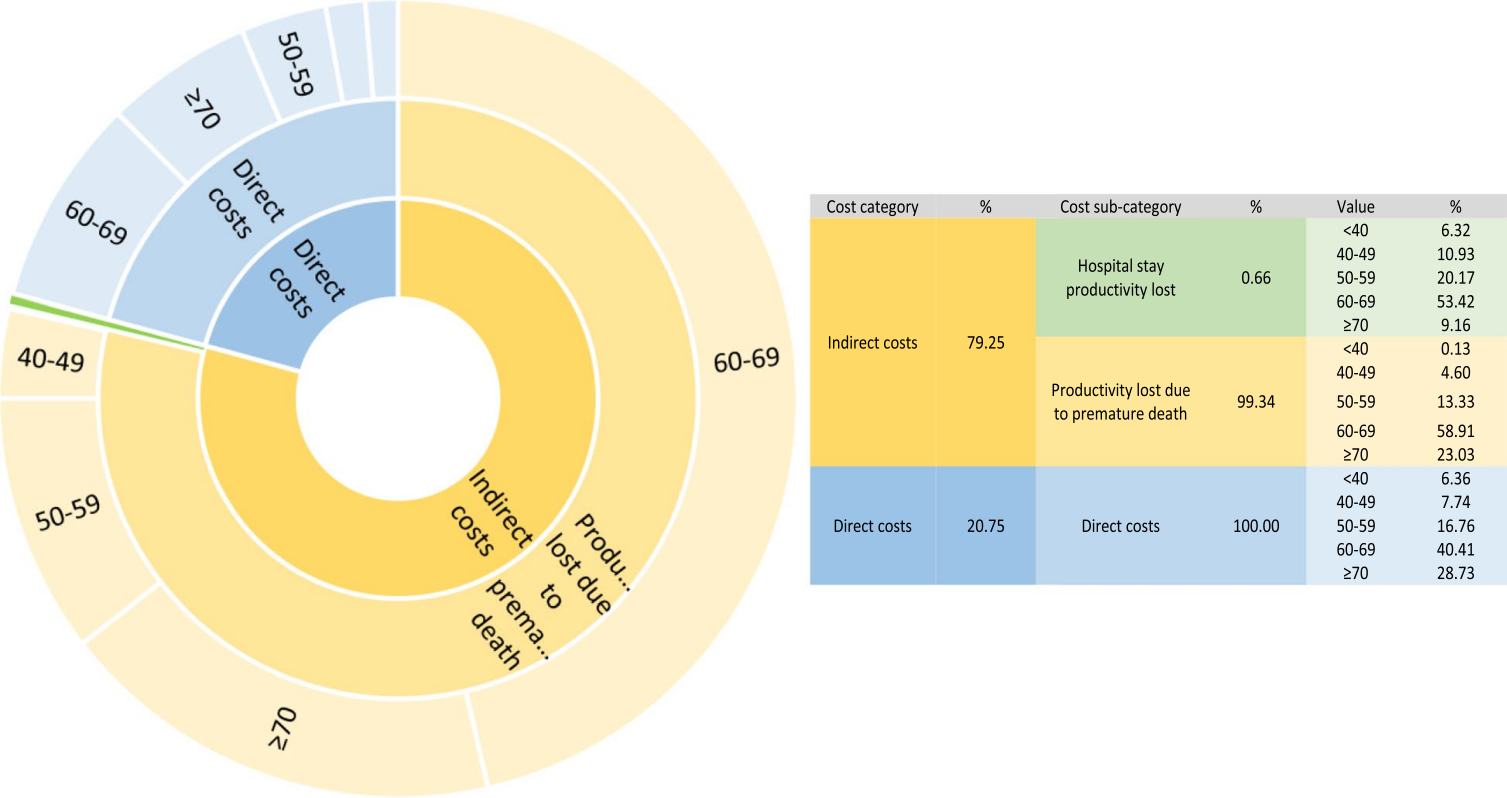
Pattern of diabetic foot ulcer costs composition

79.25% of the estimated costs (with a 5% discount rate) were indirect costs. A small percentage of indirect costs were related to productivity loss due to hospital stay (0.66%) and most of it was due to productivity loss due to premature death (99.34%). The age group of 60–69 years have borne the most expenses, so that 53.42% of the loss of productivity caused by staying in the hospital, 58.91% of the loss of productivity due to premature death and 40.41% of direct costs have been imposed on them (Fig. 1).

## Discussion

This study was conducted to estimate the economic burden of DFU in Iran. According to the estimates made, the economic burden of DFU in Iran in two scenarios of discounting future costs and not discounting them was about $8.7 billion and $35 billion, respectively. According to the reports of the World Bank, Iran's GDP in 2021 was about 1.45 trillion dollars [30], so the estimated burden is about 0.59 and 2.41% of Iran's GDP, respectively. In particular, no studies have been done to estimate the economic burden of DFU in Iran, but Jalilian's study [31] has shown that the economic burden of type 2 diabetes was about 7.7% of Iran's GDP. Since DFU is only one of the consequences of type 2 diabetes, the results of these two studies seem to be consistent.

Various approaches for estimating the economic burden of diseases have been developed, and each considers some criteria that vary between different types of costing studies. Thus, it might be misleading and biased to compare the results of cost estimations due to the substantial discrepancies and heterogeneity in studies. Nonetheless, comparing the economic burden of Iran with other countries enables us to give additional insights on the magnitude of the economic burden of diabetes mellitus [15]. The total annual direct cost for patients with DFU was estimated at over $1.8 billion and the total economic burden without discounting exceeded $35 billion. The average cost attributable to DFU in males was $2,529 and in females was $2,694. These figures concerning a gross domestic product (GDP) of US $191.7 billion and a per capita GDP of US $2282 in Iran in 2020 suggest that DFU places a heavy burden on the health system, society and economy of the country. Prevention or early diagnostic interventions targeted at high-risk populations can have specific roles in reducing the burden of DFU.

Overall, 79.25% of the estimated costs in this study were indirect costs and productivity losses, of which 99.34% were productivity losses due to premature death. The mortality rate of patients with diabetic foot ulcers in the sample of this study was 7.6%.Other studies have reported different mortality rates, for example, Jeyaraman et al. [32] reported a 5-year DFU mortality rate of 24.6% and Jupiter et al. [33] reported 40%.The results of Chen et al.'s study [34] have shown that older age, suffering from peripheral vascular disorders, kidney diseases, amputations and heart diseases are at higher risk of death due to DFU.

20.75% of the estimated costs in this study were direct costs. The mean cost per patient was $2529 for men and $2695 for women (P = not significant; Table 1). In addition, patients hospitalized with DFU were more frequently male (68.2% vs. 31.8%), these results were consistent with the findings of Lu et al. [3]. A possible explanation for this might be that women care more about their health than men do, therefore they are more likely to visit and be diagnosed early and have good compliance with standard care and treatment regimens before their health would deteriorate [35]. More than three-quarters of participants (77.3%) aged over 50 years, showing that DFU is significantly more prevalent among the older age groups, which corresponds to the findings of the previous work in this field [3]. Over half of the patients (54.3%) were in employment and were employed as drivers, general laborers, employees, etc. Total costs differed significantly between economically active and inactive patients. That is, the working population seems to be more concerned about their health and time lost from work while being treated in order to return to work as soon as possible. Hence, they may leave hospitals against medical advice so that they are more likely to be low utilizers of hospital resources.

Of the 541 patients interviewed, 97.2% were Iranian and the rest were Afghan, however, there was no significant difference in average total cost between them, implying equities in access to health services. For several decades, the people of Afghanistan have been suffering from numerous severe economic and social problems. Therefore, this country has become one of the main sources of refugees around the world, and many of its people live in different countries [36]. According to the official reports of Iran Statistics Center in 2016, 1,583,979 Afghans have lived in Iran (about 2% of the country's total population) [37]. According to the report of the United Nations Refugee Agency, about five million Afghans are displaced abroad, of which 90 percent live in Pakistan and Iran [38]. Therefore, it is obvious that in the different types of sampling that is done from the population level in different subjects, some of the people under investigation are Afghans. It is worth noting that in this study, the aim was not to estimate the economic burden by different nationalities, but due to the high number of Afghans living in Iran, some of them have been included in this study. Because they also receive health services mainly at common prices for Iranian people, the difference in their treatment costs compared to Iranian people was not statistically significant.

Analyzes have shown that 73.3% of DFU patients admitted to the hospital were discharged and recovered after receiving services, and the mortality rate for these patients was 7.6%. We found a significant association between hospital costs and patient discharge states. The highest cost of DFU treatment belonged to patients who died after receiving the service. This difference can be explained in part by the fact that progressively sicker patients receive greater ulcer-related hospital care to survive. On the other hand, patients discharged against medical advice incurred the lowest hospital costs. A possible explanation for this might be that these patients have preferred to be treated at home and leave the hospital, so the length of stay in the hospital and ultimately the services consumed by them will decrease and lead to reduced costs. In this study, total in-hospital cost is increasing in length of stay. These results match those observed in earlier studies [39, 40]. Hospital length of stay is one of the main determinants of diabetic foot ulcer costs. Increased length of stay and longer duration of treatment can result in consuming extra services like medicine, consumables, and hoteling, as a result, increases the cost of foot ulcer care. The direct cost of DFU treatment worldwide is expected to reach $74 billion [41] and its complications are increasing in particular in the Middle East [15]. It is estimated that the number of diabetic patients in Iran, one of the Middle East countries, will reach 9.24 million people by 2030 [15]. Many of the complications of the disease are not life-threatening and are generally ignored by patients, especially in developing countries, until the symptoms become severe and require care. Delays in diagnosis, quality of care, insufficient awareness, and inequality in income are the principal contributors affecting the receipt of services in developing countries [42, 43]. DFU as one of the serious complications of diabetes mellitus manifests itself when the progression of the disease occurred, therefore, patients need more specialized care. Also, some patients' feet are amputated, which results in work-off days and lost production accompanied by direct costs.

Despite the differences in the prevalence of DFU in different regions of the world, patients have a specific path to ulceration. Ulcers are mainly the result of diabetic peripheral neuropathy and peripheral vascular diseases [44]. Some preventive strategies include annual diabetic foot screening, and facilitated diabetic foot care interventions through multidisciplinary teams. These measures can have a significant effect on improving patient outcomes through early diagnosis of diabetic patients at risk of ulceration [45]. The 5 main elements in preventing DFU are: Identification of patients at risk of DFU, Regular examination of feet at risk of DFU, Educating patients, families and health care professionals, Wear appropriate footwear and Timely treatment of risk factors for ulceration [44].

### Policy implications

Considering the significant economic burden of diabetic foot ulcer and the possibility of worsening its economic and social consequences as a result of increasing the prevalence of diabetes in the coming years, policy makers should recognize it as an important health problem and plan a set of measures to control it. Some points that should be considered in the planning process for its management are:

- Prevention of diabetes is the most basic possible measure to avoid conditions resulting from diabetes such as diabetic foot ulcers. Out of 5,379,252 diabetic patients in Iran in 2019, 5,035,011 people (93.6%) had type 2 diabetes [22], so it seems that community-based measures to manage type 2 diabetes can have a significant effect on preventing foot ulcers. The causes of diabetes can be divided into two major categories: non-modifiable risk factors (genetic) and modifiable risk factors (Overweight and obesity, Sedentary lifestyle, Previously identified glucose intolerance, Metabolic syndrome such as Hypertension, Decreased HDL cholesterol, Increased triglycerides, Dietary factors, Intrauterine environment and Inflammation) [46]. To prevent type 2 diabetes, a set of complementary clinical and public health strategies should be done. The roles of the public health sector include: monitoring diabetes risk, establishing a diabetes prevention program with the participation of various community groups, ensuring the quality of prevention programs, developing and implementing appropriate policies to reduce diabetes risk by facilitating lifestyle modification and improving the community environment in a way that makes healthy behaviors easier. The clinical department can be involved in identifying risk status, referring high-risk people to community-based lifestyle modification programs, providing nutritional counseling, prescribing medication when necessary, timely treatment, and preventing the development of the disease [47].
- According to the global burden of diseases reports, the prevalence of diabetes per 100,000 people in Iran has varied from about 2,580 in Sistan and Baluchistan to about 8,370 in South Khorasan [22]. Therefore, for a more efficient management of diabetes and its complications, including diabetic foot ulcers, it is necessary to first assess the current situation of the regions in terms of the prevalence of diabetes and its consequences, and predict its future trends and then, taking into account the needs of each region, plan for preventive and therapeutic measures. This approach can be an effective step in increasing the cost-effectiveness of the disease management program.
- Financial insecurity can have a major impact on the prognosis of wounds [48]; therefore, it is necessary to ensure that the required procedures and drugs are available to those in need of care with insurance coverage and minimal patient cost-sharing.

### Strengths and limitations

The key strength of the present study is its large sample size drawn from the metropolis of Tehran, a nationally representative sample of the Iranian population. Despite the mentioned strengths, this study also has some limitations, First, since the study was retrospective, data for some economic and social variables were not available. Secondly, the study did not evaluate intangible costs on the ground that the quantification of these types of costs is a thorny issue. Third, Due to the lack of access to the required data, it was not possible to estimate the costs and economic burden based on the severity of the DFU.

## Conclusion

DFU, as a chronic and long-term complication of diabetes, imposes a significant economic burden on the health system and society, so that the growing trend of diabetes can turn it into an important socio-economic problem in the coming decades. Therefore, identification of vulnerable populations in different regions of the country, adoption of preventive and therapeutic strategies in accordance with the epidemiology of the disease in each region should be pursued seriously. These strategies can include a combination of public and clinical health interventions in preventing the occurrence of diabetes or preventing its development as an underlying disease and the primary source of the possibility of diabetic foot ulcers. These strategies can include a combination of public and clinical health interventions in preventing the occurrence of diabetes or preventing its development as an underlying disease and the primary source of the possibility of diabetic foot ulcers. At the same time, educating diabetic patients; Creating the necessary mechanisms to monitor diabetic patients in terms of various types of neuropathies and underlying disorders of ulceration, providing clinical care using multidisciplinary teams for wound management, including specialized wound clinics, and ensuring patients' access to care necessary by providing insurance coverage for health services provided in these specialized clinics.

## Data Availability

The data used for analysis for the current study are not publicly available due to privacy limitations but are available from the corresponding author upon reasonable request.
